# Novel *FRMD6::PTH* chimera in tumorous bone lesion carrying a t(4;11;14;12)(q35;p15;q22;q13)

**DOI:** 10.3389/pore.2025.1612096

**Published:** 2025-06-26

**Authors:** Ioannis Panagopoulos, Kristin Andersen, Isabel Lloret, Ludmila Gorunova, Ingvild Lobmaier

**Affiliations:** ^1^ Section for Cancer Cytogenetics, Institute for Cancer Genetics and Informatics, The Norwegian Radium Hospital, Oslo University Hospital, Oslo, Norway; ^2^ Department of Molecular Oncology, Institute for Cancer Research, The Norwegian Radium Hospital, Oslo University Hospital, Oslo, Norway; ^3^ Department of Radiology, The Norwegian Radium Hospital, Oslo University Hospital, Oslo, Norway; ^4^ Department of Pathology, The Norwegian Radium Hospital, Oslo University Hospital, Oslo, Norway

**Keywords:** fibro-osseous tumor, chromosomal translocation, FERM domain containing 6 (FRMD6), parathyroid hormone (PTH), *FRMD6::PTH* chimera

## Abstract

**Background:**

Benign fibro-osseous lesions are characterized by the replacement of normal bone with cellular fibrous connective tissue with new bone formation. The published cytogenetic information on these tumors is limited to only few cases. Here, we report the cytogenetic and molecular genetic findings of a fibro-osseous tumor.

**Methods:**

A fibro-osseous lesion was investigated for genetic abnormalities using banding cytogenetics, fluorescence *in situ* hybridization (FISH), RNA sequencing, and direct cycle Sanger sequencing.

**Results:**

The karyotype was 46,XX,t(4;11;14;12)(q35;p15;q22;q13)[7]/46,XX [3], with no rearrangement of *HMGA2*. RNA sequencing revealed two *FRMD6::PTH* chimeric transcripts, originating from the fusion point 14q22;11p15 of the t(4;11;14;12). In these transcripts, exon 1 of *FRMD6* fused to either exon 1 or exon 2 of *PTH*. Direct cycle sequencing confirmed the presence of these *FRMD6::PTH* chimeric transcripts.

**Conclusion:**

This study reports, for the first time, the presence of the *FRMD6::PTH* chimera in fibro-osseous tumor. In this chimera the expression of the entire coding region of *PTH* is regulated by the ubiquitously expressed *FRMD6* gene promoter. Dysregulation of *PTH* expression may have significant implications for processes regulated by PTH protein.

## Introduction

Benign fibro-osseous tumors of bone are neoplasms in which normal bone is replaced by cellular fibrous connective tissue with areas of new bone formation within the fibrous stroma [1, 2]. They are among the most common benign bone lesions in children and adolescents and represent a group of clinically distinct entities with significant histologic overlap [1, 2]. Although the exact cause of these tumors is unknown, they are believed to result from disruptions in the normal process of bone formation and remodeling [1, 2]. Fibro-osseous tumors occur in various body locations, including the skull, jawbones, and long bones, and typically exhibit slow growth over time. They encompass several types such as fibrous dysplasia, ossifying fibroma, osteo-fibrous dysplasia, and cemento-osseous dysplasia, which can lead to diagnostic challenges due to overlapping clinical, radiological, and pathological features [1, 2].

Published cytogenetic information on benign fibro-osseous tumors of bone is limited to few cases only [3, 4]. However, recent studies have suggested that diagnostic genetics may serve as an ancillary use in evaluating these tumors [1, 2]. Molecular genetic investigations have shown that fibro-osseous tumors can be associated with specific genetic alterations, which may aid in differential diagnosis. For example, in fibrous dysplasia somatic variants at codon 201 or codon 227 of the GNAS complex locus (*GNAS* on chromosomal sub-band 20q13.32) have been reported with frequencies ranging from 45% to 100% [5, 6]. These variants substitute arginine at codon 201 (p.R201) or glutamine at position 227 (p.Q227) resulting in constitutive activation of GNAS protein [5, 6]. Similarly, psammomatoid ossifying fibroma has been associated with rearrangements of the SATB homeobox 2 gene (*SATB2* on chromosomal sub-band 2q33.1), as identified through whole transcriptome sequencing [7]. Moreover, a combination of whole exome sequencing and RNA sequencing has revealed fusion transcripts and variants in many genes in cemento-ossifying fibroma [8].

In the present study we applied a sequential approach combining karyotyping and RNA-sequencing to investigate a fibro-osseous tumor. Our aim was to identify neoplasm-specific fusion genes that could contribute to its pathogenesis.

## Case presentation

**FIGURE 1:**
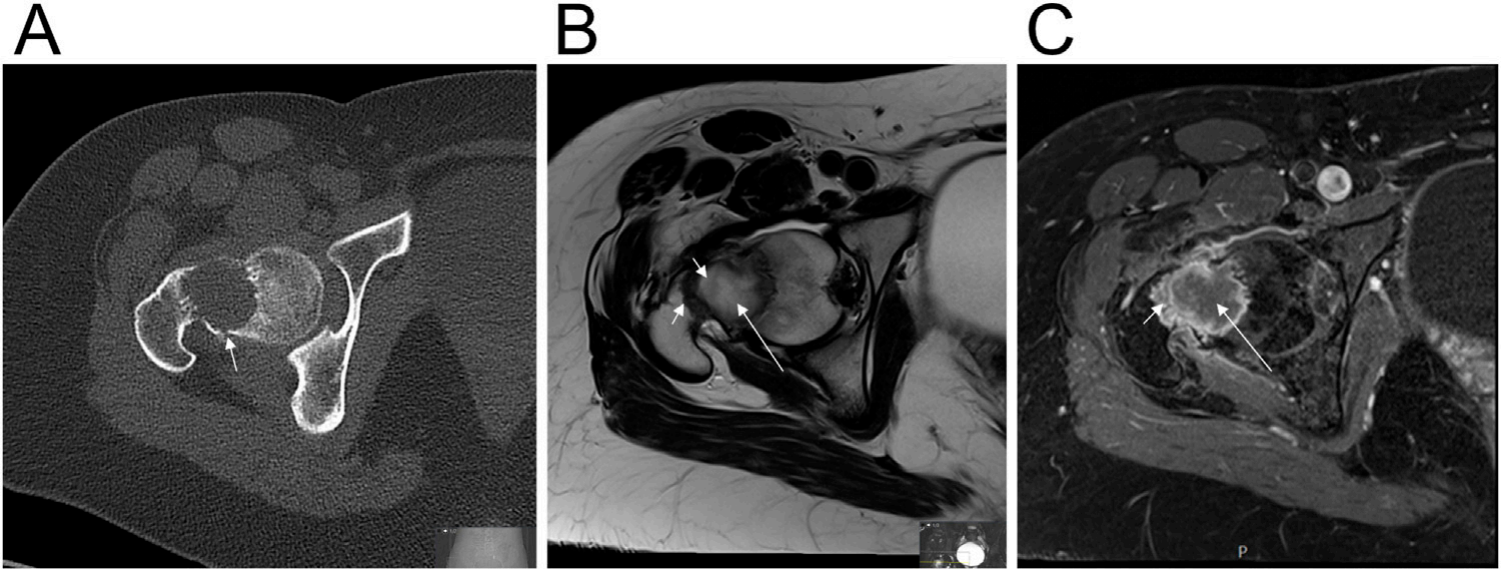
Radiological features of the fibro-osseous tumor. **(A)** Axial CT image shows a well-defined osteolytic lesion in the femoral neck with a pathological fracture (arrow) and no visible mineralization. **(B)** Axial T2-weighted MRI of the lesion shows a central part with high signal intensity which may represent water content (long arrow), and a peripheral border as well as intralesional stripes, both with low signal intensity, which may correspond to fibrous content (short arrows). **(C)** Axial Fat-suppressed contrast-enhanced T1-weighted MRI of the lesion reveals that the central part doesn’t have contrast enhancement which representing water content (long arrow). The peripheral border has contrast enhancement indicating vascularized tissue (short arrow).

A 23-year-old woman presented with a 6-month history of pain in her right hip. The pain significantly worsened after minor trauma to the hip. She required the use of crutches and was admitted to a local hospital, where X-ray, computed tomography (CT), and magnetic resonance imaging (MRI) were performed. There were no previous imaging studies available for comparison. X-ray and CT revealed a well-defined osteolytic lesion in the femoral neck with a pathological fracture and no visible mineralization (Figure 1A). MRI showed that the lesion had a central area with high T2 signal intensity and high diffusion but no contrast enhancement, suggesting water content (Figures 1B,C). The lesion also had a peripheral border and intralesional stripes, both showing low T2 signal, which may represent fibrous content (Figure 1B). Contrast enhancement was seen along the peripheral border, indicating vascularized tissue (Figure 1C). Radiologically, the lesion had a benign appearance. Based on its location and signal pattern, cystic fibrous dysplasia or a simple bone cyst was suspected. The patient was treated with curettage of the lesion, packing with allogenic bone graft, and plate osteosynthesis.

**FIGURE 2:**
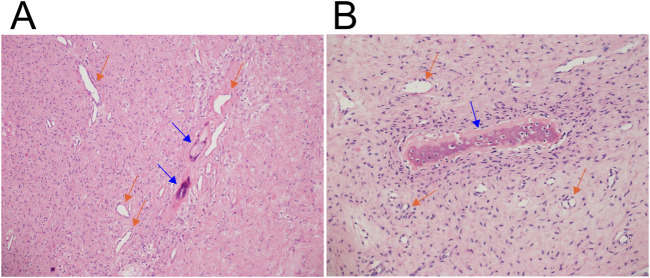
Microscopic examination of the fibro-osseous tumor. Hematoxylin and eosin-stained sections show fibrous tissue lacking cytological atypia, with rich vascularization (orange arrows), and areas of loosely organized stroma. Scattered foci of new bone formation (blue arrows), lacking osteoblastic rimming, are also observed. **(A)** Magnification ×100. **(B)** Magnification ×200.

Microscopic examination revealed a lesion composed of both fibrous and osseous components (Figures 2A,B). The fibrous tissue lacked cytological atypia and was partially loosely organized. The osseous component consisted of scattered foci of new bone formation, lacking osteoblastic rimming. Immunohistochemical analysis demonstrated negative staining for S100 protein, desmin, ACTA2 (also known as alpha-smooth muscle actin, α-SMA), and CD34 (data not shown). The findings were consistent with a benign fibro-osseous lesion, although a more specific classification was not established.

**FIGURE 3:**
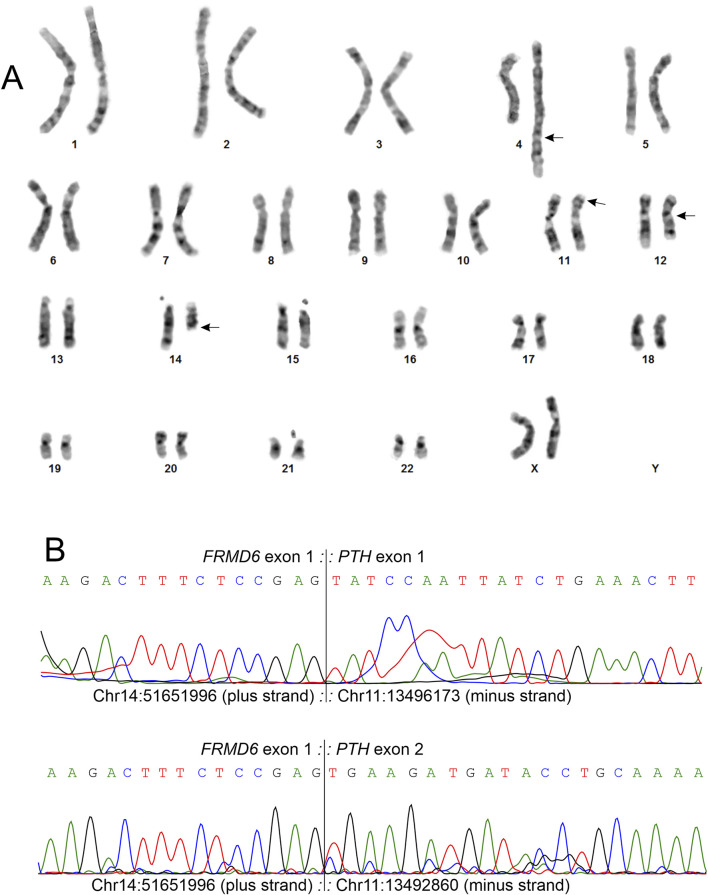
Genetic examination of the fibro-osseous tumor. **(A)** Karyogram showing the aberrant chromosomes 4, 11, 12, and 14 involved in a complex four-way translocation t(4;11;14;12)(q35;p15;q22;q13), along with their corresponding normal homologs. Breakpoint positions are indicated by arrows. **(B)** Partial Sanger sequencing chromatograms demonstrating the *FRMD6::PTH* fusion point, where exon 1 of the *FRMD6* gene (transcript variant 2, NM_152330.4) is fused to exons 1 and 2 of the *PTH* gene (transcript variant 2, NM_001316352.2). The exact fusion point coordinates, based on the GRCh38/hg38 assembly, are chr14: 51651996 (plus strand) for *FRMD6* exon 1 and chr11:13496173 and for chr11:13492860 (both minus strand) for *PTH* exons 1 and 2, respectively. The two fusion transcript sequences have been submitted to the GenBank database and assigned the accession numbers PV085792 and PV085793.

G-banding analysis of tumor cells [9] detected a four-way translocation involving chromosomal bands 4q35, 11p15, 14q22, and 12q13 as the sole cytogenetic aberration in 7 out of 10 examined metaphases (Figure 3A). The resulting abnormal karyotype was 46,XX,t(4;11;14;12)(q35;p15;q22;q13)[7]/46,XX[3]. FISH analysis on 96 interphase nuclei, using an in-house prepared *HMGA2* break-apart probe as previously described [10], did not detected rearrangement of the *HMGA2* gene (data not shown).

Total RNA extracted from frozen (−80°C) tumor tissue adjacent to that used for cytogenetic and histological analyses underwent high-throughput paired-end sequencing at the Genomics Core Facility, Norwegian Radium Hospital, Oslo University Hospital. Using FusionCatcher software [11] with fastq files from RNA sequencing, two *FRMD6::PTH* chimeric transcript sequences were detected, corresponding to the fusion points 14q22; 11p15 of the aforementioned four-way translocation. In the first transcript, exon 1 of *FRMD6* fused to exon 1 of PTH: 5′-AGA GGG GTG ACC AGA GAG CCC AAC GCC TGG TGC TCA AGA CTT TCT CCG AG::TA TCC AAT TAT CTG AAA CTT AAG AAG AGT GTG CAC CGC CCA ATG GGT GTG-3´. In the second *FRMD6::PTH* chimeric transcript, exon 1 of *FRMD6* fused to exon 2 of *PTH*: 5′-GTG ACC AGA GAG CCC AAC GCC TGG TGC TCA AGA CTT TCT CCG AG::TG AAG ATG ATA CCT GCA AAA GAC ATG GCT AAA GTT ATG ATT GTC-3′.

To confirm the presence of the fusion transcripts, complementary DNA (cDNA) was synthesized from 400 ng of total RNA and cDNA corresponding to 20 ng of total RNA was used as the template in the subsequent PCR/cycle (Sanger) assays using the BigDye Direct Cycle Sequencing Kit, following the manufacturer’s recommendations (ThermoFisher Scientific, Waltham, MA, United States). The primer combinations were FRMD6-13F1 (5′-AGG CTC GGC GCC GGT AGG AA-3′)/PTH-40R1 (5′-CAC ACA CCC ATT GGG CGG TGC-3′) and FRMD6-26F1 (5′- GTA GGA AGA GTC AGA GGG GTG ACC A-3′)/PTH-204R1 (5′-AAC AGA TTT CCC ATC CGA TTT TGT AA-3′). The *FRMD6* forward primers had the M13 forward primer sequence (TGT​AAA​ACG​ACG​GCC​AGT) at their 5′-end, and the *PTH* reverse primers had the M13 reverse primer sequence (CAG​GAA​ACA​GCT​ATG​ACC) at their 5′-end. Sequence analysis was performed using the Applied Biosystems SeqStudio Genetic Analyzer (ThermoFisher Scientific), and the obtained sequencing data were aligned with the reference sequences NM_152330.4 for *FRMD6* and NM_001316352.2 for *PTH* using the Basic Local Alignment Search Tool (BLAST). Direct cycle sequencing confirmed the presence of both *FRMD6::PTH* transcripts, as shown in Figure 3B. The two fusion transcript sequences have been submitted to the GenBank database and have been assigned the accession numbers PV085792 and PV085793. The Supplementary Data Sheet provides detailed descriptions of the methodologies used for G-banding and karyotyping, fluorescence in situ hybridization (FISH), RNA sequencing, and molecular genetic analyses.

## Discussion

The *FRMD6* gene (located at sub-band 14q22.1) encodes the 4.1-ezrin-radixin-moesin (FERM) domain-containing protein 6 (FRMD6, also known as Willin) [12, 13]. This protein has diverse cellular functions, including the regulation of the Hippo and mTOR signaling pathways [13]. *FRMD6* has four transcript variants (accession numbers NM_001042481.3, NM_001267046.2, NM_001267047.1, and NM_152330.4) and is broadly expressed across multiple tissues [12, 13]. In these transcripts, either exon 1 (NM_001267046.2, NM_001267047.1, and NM_152330.4) or exons 1 and 2 (NM_001042481.3) are untranslated. Chimeras involving *FRMD6* as the 5′-end partner gene have been reported in less than ten tumors [14, 15] (Table 1). In all these cases, the untranslated exons of *FRMD6* fused to the 3′-end partner gene, with the promoter and other 5′-end regulatory elements of *FRMD6* driving the expression of the 3′-end partner gene [16, 17].

**TABLE 1:** Published cases in which chimeric genes have *FRMD6* as the 5′-end partner and cases in which chimeric genes have *PTH* as 3′-end partner.

Tumor type	Chimera	Reference
head and neck squamous cell carcinoma	*FRMD6* [NM_001042481.3] exon 2::*BPIFB1* [NM_033197.3] exon 2	[14]
mesothelioma	*FRMD6* [NM_152330.4] exon 1::*GNG2* [NM_053064.5] exon 3	[14]
bladder urothelial carcinoma	*FRMD6* [NM_001042481.3] exon 2::RTN4 [NM_007008.3] exon 2	[14]
lung adenocarcinoma	*FRMD6* [NM_152330.4] exon 1::*SCFD1* [NM_016106.4] exon 15	[14]
salivary duct carcinoma	*FRMD6* [NM_152330.4] exon 1::*PLAG1* [NM_002655.2] exon 3	[15]
lung squamous cell carcinoma	*BTBD10* [NM_032320.7] exon 1::*PTH* [NM_001316352.1] exon 1	[14]
breast invasive carcinoma	*NUMA1* [NM_006185.4] exon 1::*PTH* [NM_001316352.1] exon 1	[14]
fibro-osseous tumor	*FRMD6* [NM_152330.4] exon 1::*PTH* [NM_001316352.2] exon 1 *FRMD6* [NM_152330.4] exon 1::*PTH* [NM_001316352.2] exon 2	Present study

The *PTH* gene (located at sub-band 11p15.2) is exclusively expressed in the parathyroid glands, where it encodes parathyroid hormone (PTH) [18, 19]. This hormone plays a crucial role in regulating blood calcium levels, as well as in phosphorus metabolism, bone formation, and resorption [18, 19]. The expression of the *PTH* gene is regulated by numerous transcription factors, including both activators and repressors [20, 21]. A 5.2-kbp DNA region immediately upstream of the human *PTH* gene was found to be sufficient in order to drive parathyroid-specific gene expression [21]. Ectopic expression of *PTH* by tumor cells has been reported in fewer than 30 cases, often contributing to hypercalcemia associated with malignancy [22]. In an ovarian carcinoma the expression was linked to rearrangement of the *PTH* gene [23], while in a case of high-grade neuroendocrine carcinoma of the pancreas, ectopic *PTH* expression was associated with hypomethylation of the *PTH* locus [24]. In other non-parathyroid tumors, however, ectopic *PTH* expression was not associated with rearrangement, amplification or hypomethylation of the *PTH* gene [22]. Chimeric transcripts with *PTH* as the 3′-end partner gene have been described in two tumors: in a lung squamous cell carcinoma, where exon 1 of the BTB domain containing 10 (*BTBD10*) gene fused to exon 1 of *PTH* (*BTBD10::PTH*) and in a breast invasive carcinoma, where exon 1 of the nuclear mitotic apparatus protein 1 (*NUMA1*) fused to exon 1 of *PTH* (*NUMA1::PTH*) [14]. These chimeric transcripts indicate genomic rearrangements involving both *PTH* and the respective 5′-end partner genes, suggesting that the promoters and other 5′-end regulatory elements of the ubiquitously expressed *BTBD10* or *NUMA1* control *PTH* expression.

To the best of our knowledge, the *FRMD6::PTH* chimera, identified in the examined fibro-osseous tumor, is reported for the first time in this study. The *FRMD6::PTH* chimera is predicted to be located on the der (14) chromosome resulting from the four-way translocation, as both the *PTH* gene (located on 11p15.2) and the *FRMD6* gene (located on 14q22.1) are transcribed from centromere to telomere. The *FRMD6::PTH* chimeric transcript follows a pattern observed in previous reported chimeras involving *FRMD6* as the 5′-end partner gene, where the untranslated exons of *FRMD6* fused to the 3′-end partner gene. It also exhibits the pattern found in the two previously described chimeric transcripts with *PTH* as the 3′-end partner gene, involving the fusion of the entire translated part of *PTH*, with its expression controlled by the promoter and other 5′-end regulatory elements of ubiquitously expressed genes. Dysregulation of *PTH* expression may have implications for calcium homeostasis and other processes regulated by PTH protein.

Although the presence of the chimeric transcript strongly suggests transcriptional activation of *PTH* in the tumor, the actual expression of functional PTH protein and activation of downstream signaling pathways remain speculative. Potential regulatory mechanisms may include promoter swapping, whereby the promoter of *FRMD6* -a gene expressed in a wide range of tissues-drives ectopic expression of *PTH*, as well as changes in chromatin structure or local epigenetic alterations induced by the translocation event [25, 26]. Moreover, the preservation of the full *PTH* coding sequence in the chimera raises the possibility that biologically active hormone could be produced, although this has not yet been demonstrated.

Importantly, PTH is not only a regulator of calcium metabolism but also plays a role in bone remodeling by stimulating both osteoclastic bone resorption and osteoblastic bone formation [27, 28]. During these remodeling processes, fibrous tissue may form as part of the bone repair response, especially in areas undergoing resorption. PTH has also been shown to enhance vascularization within bone by promoting microvessel formation [29], which may support the development of a fibrous stroma. These biological effects suggest that aberrant *PTH* expression in tumor cells could contribute to the fibro-osseous characteristics of the tumor, potentially by promoting fibrous matrix production, increased vascularity, and altered bone metabolism.

Future functional studies are needed to explore the biological significance of this fusion. These should include analyses at the transcript and protein levels to confirm PTH expression, for example using qRT-PCR, western blotting, or immunohistochemistry. Assays to evaluate PTH1R-mediated signaling, such as measurement of cAMP accumulation or phosphorylation of downstream effectors, could help determine whether the PTH pathway is functionally active [30, 31]. *In vitro* expression of the *FRMD6::PTH* fusion construct in model cell lines would provide further insight into its biological role and could clarify whether the fusion promotes tumor cell proliferation, survival, osteogenic differentiation, or fibrous tissue formation, as has been shown for other oncogenic fusion genes [32]. Such investigations would provide valuable insights into whether this fusion plays a direct oncogenic role or represents a passenger alteration.

## Conclusion

This study reports the first fibro-osseous tumor carrying the *FRMD6::PTH* chimeric gene, resulting from a chromosomal aberration. This fusion potentially disrupts *PTH* expression, warranting further investigation into its role in tumorigenesis.

## Data Availability

The original contributions presented in the study are included in the article/Supplementary Material, further inquiries can be directed to the corresponding author.
