# Supplementary material for: Novel FRMD6::PTH chimera in tumorous bone lesion carrying a t(4;11;14;12)(q35;p15;q22;q13)
Source: Pathol Oncol Res. 2025 Jun 26;31:1612096. doi: 10.3389/pore.2025.1612096 (PMC12240868; doi:10.3389/pore.2025.1612096)
Supplement: Supplementary file 1 [file DataSheet1.pdf]

Methods

G-Banding and karyotyping

A part of the resected tumor specimen was minced into 1–2 mm fragments using scalpels and enzymatically disaggregated with collagenase II (Worthington, Freehold, NJ, USA). The resulting cells were cultured, harvested, and processed for chromosome examination following standard cytogenetic protocols [1]. Chromosome G-banding was performed using Wright's stain (Sigma Aldrich; St Louis, MO, USA). Metaphase spreads were analyzed, and karyograms were prepared using the CytoVision computer-assisted karyotyping system (Leica Biosystems, Newcastle upon Tyne, UK). The karyotype was described according to the International System for Human Cytogenomic Nomenclature (ISCN 2020) [2].

Fluorescence in situ hybridization (FISH)

FISH analysis was performed on interphase nuclei using an in-house prepared *HMGA2* break-apart probe as previously described [3]. The probe was made from commercially available bacterial artificial chromosomes (BAC) purchased from the BACPAC Resource Center operated by BACPAC Genomics, Emeryville, CA, USA (https://bacpacresources.org/).

The FISH probes were prepared from BAC DNAs amplified with bacteriophage Phi29 DNA polymerase, using previously described methodology [4] and commercial kits for DNA isolation, amplification, labelling, and hybridization, according to the

manufacturers' instructions. In brief, single bacterial colonies were grown overnight in 5 ml culture and BAC DNA was purified using High Pure Plasmid Isolation Kit (Roche Diagnostics, Mannheim, Germany). Following purification, BAC DNAs were isothermally amplified using the GenomiPhi V2 DNA Amplification Kit (Cytiva, Marlborough, Massachusetts, United States). The amplified BAC DNAs were then labeled using Abbott's nick translation kit (Abbott Molecular, Des Plaines, IL, USA) and hybridized to the chromosome preparations.

All BAC clones map to chromosome sub-band 12q14.3 and span the *HMGA2* locus. Their positions on the GRCh38/hg38 assembly are as follows:

RP11-185K16: Chr12:65427017-65594623; RP11-30I11: Chr12:65498459-65669659; RP11-662G15: Chr12:65608717-65818177; RP11-118B13: Chr12:65964922-66109206; RP11-745O10: Chr12:66083023-66208799; and RP11-263A04: Chr12:66246378-66412442.

The centromeric (proximal) part of the probe was constructed from a pool of clones RP11-185K16, RP11-30I11, and RP11-662G15, and labelled with Texas Red-5-dCTP (PerkinElmer, Boston, MA, USA) to produce a red signal. The telomeric (distal) part of the probe was constructed from a pool of clones RP11-118B13, RP11-745O10, and RP11-263A04, and labelled with fluorescein-12-dCTP (PerkinElmer) to produce a green signal. Chromosome preparations were counterstained with 0.2 μg/ml DAPI and overlaid with a 24 x 50 mm2 coverslip. Fluorescent signals were captured and analyzed using the CytoVision system (Leica Biosystems).

RNA sequencing

Total RNA was extracted from frozen (-80 °C) tumor tissue adjacent to that used for cytogenetic analysis and histological examination, using the miRNeasy Mini Kit and Qiacube system (Qiagen, Hilden, Germany). RNA concentration was measured with a QIAxpert microfluidic UV/VIS spectrophotometer (Qiagen). RNA integrity was assessed using an Agilent 2100 Bioanalyzer. The DV200 index, which evaluates the percentage of RNA fragments longer than 200 nucleotides, was found to be 70% [5]. A total of 200 ng of RNA was sent to the Genomics Core Facility at the Norwegian Radium Hospital, Oslo University Hospital, for high-throughput paired-end RNA sequencing. The FusionCatcher software was used to identify potential fusion transcripts [6, 7].

Molecular genetic investigation

Complementary DNA (cDNA) was synthesized from 400 ng of total RNA, and cDNA corresponding to 20 ng of total RNA was used as template in the subsequent PCR assay. The BigDye Direct Cycle Sequencing Kit (ThermoFisher Scientific, Waltham, MA, USA) was used for PCR and Sanger sequencing according to the manufacturer's recommendations. The primer combinations used were: FRMD6-13F1 (5´-AGG CTC GGC GCC GGT AGG AA-3´)/PTH-40R1 (5´-CAC ACA CCC ATT GGG CGG TGC-3´) and FERM-26F1 (5´- GTA GGA AGA GTC AGA GGG GTG ACC A -3´)/PTH-204R1 (5´-AAC AGA TTT CCC ATC CGA TTT TGT AA-3´). The *FRMD6* forward primers included the M13 forward primer sequence (5′-TGT AAA ACG ACG GCC AGT-3′) at their 5′ end, while the *PTH* reverse primers included the M13 reverse primer sequence (5′-CAG GAA

ACA GCT ATG ACC-3′) at their 5′ end. Sequence analyses were carried out using the Applied Biosystems SeqStudio Genetic Analyzer system (ThermoFisher Scientific). The obtained sequences were compared to reference sequences NM_152330.4 (*FRMD6*) and NM_001316352.2 (*PTH*) using the Basic Local Alignment Search Tool (BLAST) [8] and aligned to the GRCh38/hg38 assembly using the Genome Browser at the University of California, Santa Cruz (UCSC) [9, 10].

References

1. Lukeis R, Suter M. Cytogenetics of solid tumours. *Methods Mol Biol* (2011) 730:173-87. doi: 10.1007/978-1-61779-074-4_13
2. McGowan-Jordan J, Hastings RJ, Moore S. ISCN 2020: An International system for human cytogenomic nomenclature (2020) Basel: Karger; 2020.
3. Panagopoulos I, Gorunova L, Andersen K, Lund-Iversen M, Hognestad HR, Lobmaier I, et al. Chromosomal translocation t(5;12)(p13;q14) leading to fusion of high-mobility group at-hook 2 gene with intergenic sequences from chromosome sub-band 5p13.2 in benign myoid neoplasms of the breast: A second case. *Cancer Genomics Proteomics* (2022) 19(4):445-55. doi: 10.21873/cgp.20331
4. Roohi J, Cammer M, Montagna C, Hatchwell E. An improved method for generating BAC DNA suitable for FISH. *Cytogenet Genome Res* (2008) 121(1):7-9. doi: 10.1159/000124374
5. Matsubara T, Soh J, Morita M, Uwabo T, Tomida S, Fujiwara T, et al. DV200 Index for Assessing RNA Integrity in Next-Generation Sequencing. *Biomed Res Int* (2020) 2020:9349132. doi: 10.1155/2020/9349132
6. Kangaspeska S, Hultsch S, Edgren H, Nicorici D, Murumagi A, Kallioniemi O. Reanalysis of RNA-sequencing data reveals several additional fusion genes with multiple isoforms. *PLoS One* (2012) 7(10):e48745. doi: 10.1371/journal.pone.0048745
7. Nicorici D, Satalan H, Edgren H, Kangaspeska S, Murumagi A, Kallioniemi O, et al. FusionCatcher – a tool for finding somatic fusion genes in paired-end RNA-sequencing data. *bioRxiv* (2014). doi: DOI:10.1101/011650
8. Altschul SF, Gish W, Miller W, Myers EW, Lipman DJ. Basic local alignment search tool. *J Mol Biol* (1990) 215(3):403-10. doi: 10.1016/S0022-2836(05)80360-2
9. Kent WJ. BLAT--the BLAST-like alignment tool. *Genome Res* (2002) 12(4):656-64. doi: 10.1101/gr.229202
10. Kent WJ, Sugnet CW, Furey TS, Roskin KM, Pringle TH, Zahler AM, et al. The human genome browser at UCSC. *Genome Res* (2002) 12(6):996-1006. doi: 10.1101/gr.229102
